# Apology Isn't Good Enough: An Apology Suppresses an Approach Motivation but Not the Physiological and Psychological Anger

**DOI:** 10.1371/journal.pone.0033006

**Published:** 2012-03-22

**Authors:** Kenta Kubo, Kazuo Okanoya, Nobuyuki Kawai

**Affiliations:** 1 Okanoya Emotional Information Project, Exploratory Research fpr Advanced Technology (ERATO), Japan Science and Technology Agency, Nagoya, Japan; 2 RIKEN Laboratory, Brain Science Institute, Wako, Japan; 3 Department of Cognitive and Behavioral Sciences, The University of Tokyo, Meguro, Japan; 4 Department of Cognitive Science, Nagoya University, Nagoya, Japan; The University of South Wales, Australia

## Abstract

Although studies have emphasized the multiple components of anger, little is known about the physiological and psychological mechanisms of the approach motivational component and the negative emotional component of anger. In the present study, participants wrote brief opinions about social problems (e.g., tuition hikes) and received a handwritten, insulting comment about their composition from the experimenter. Half of the participants (apology group) received a simple apologetic sentence at the end of the insulting comment. Half of the participants (no apology group) did not receive one. The physiological responses of the participants were recorded prior to, and after they read the comments. Increases in heart rate and asymmetric frontal brain activity were suppressed only in the apology group. Both groups showed an increase in skin conductance response. Our psychological scales showed that the apology suppressed self reported state anger from an approach-motivational standpoint but not from a negative emotional standpoint. The results suggest that anger is not a unitary process but has multiple components. The apology did provide a different physiological profile but did not dampen down the subjective experience of anger. Thus, providing an apology may not always be effective for alleviating the experience of anger to an insult.

## Introduction

Given the consequences of actions resulting from extreme anger, the ability to successfully reduce the anger response is essential for social harmony. One common way to suppress anger is to apologize to the angered person. Although we often see people apologizing as a way to soothe anger, little is known about the efficacy of the apology and underlying mechanisms (e.g. physiological and neural) involved when an angered person has received an apology. A recent study showed that after experiencing a transgression in a trust game, people who received an actual apology were less satisfied than people who only imagined receiving one [1]. One interpretation for this result is that an apology may not be as effective in suppressing anger because the apology does not necessarily signal repentance. Perhaps, at best, it can indicate that the person giving the apology has positive qualities.

Previous research suggests that an apology is effective in reducing at least one component of anger. In one study, an experimental assistant prevented a participant from performing a task [2]. After the task, the experimenter told the participants that they performed poorly. Although participants experienced anger, those who received an apology from the assistant reported a significantly lower aggression score than those who did not receive one [2], [3]. An apology also affects the anger-elicited physiological reactions in the autonomic nervous system (ANS). When people experience anger, arousal is observed in the form of muscle tension, accelerated heartbeat, changes in breathing, and flushing in the face. These experiences are characterized by changes in ANS activity. According Ekman et al. (1983), anger produces a higher heart rate (HR), higher skin temperature, and a larger skin conductance response. These ANS patterns can be distinguished from those of other basic emotions [4]. In one study, when angered participants received a sincere apology from an adversary, anger-related high blood pressure recovered more quickly than for participants who did not receive an apology [5]. These results indicate that an apology may be effective in suppressing physiological expressions of anger. However, it is still unclear whether such a change in physiology appropriately reflects the subjective experience of anger.

Anger is said to include multiple components [6], [7]. It is thought to include not just a negative emotional component, but also an approach motivational component [8], [9], [10], [11], [12], [13]. Recent studies have emphasized the approach motivational component of anger. This component of anger has been well characterized by changes in the central nervous system (e.g., asymmetric frontal brain activity from electroencephalogram (EEG) recordings [8], [14]). One study showed that when people became angry after receiving insulting comments, they exhibited greater alpha frequency power in the right frontal area than in the left area [11]. Because alpha power is inversely related to activity, greater right frontal alpha frequency suggests greater left frontal activation [12], [13], [14], [15]. Interestingly, reducing the approach motivation of the participants can eliminate this asymmetric frontal brain activity. People in a supine body position did not show asymmetric frontal brain activity even though they read insulting comments that had been made about them. However, people sitting naturally on a chair showed asymmetric activity [16]. Another study reported the elimination of asymmetric brain activity by preventing the approach motivation of the angered person [12]. Importantly, the lack of asymmetry does not necessarily mean that people did not feel anger. They still felt anger even when they did not show asymmetric brain activity [12]. In other words, the suppression of the approach motivation of anger may not be sufficient for suppressing the subjective experience of anger [12], [16].

Very little is known about how an apology affects anger. Does an apology suppress only the negative emotional component, the motivational component, or both? Critical information is lacking on how these components relate to the physiological responses in the central nervous system and the ANS as well as to the subjective experience of anger. To our knowledge, no study has examined whether asymmetric frontal brain activity relates to ANS activity in response to anger [4]. For the current study, we recorded EEG signals, HR and skin conductance levels (SCL) and subjective measures of emotion in order to gain a better understanding of the neural and psychological mechanisms involved in an apology's influence on anger. We set up an insult situation to provoke anger in the participants [11], [16]. Half of the participants received a simple apology sentence after receiving an insulting comment (apology group). The other half of the participants did not receive the apology (no apology group). To dissociate the approach motivational component and the negative emotional component of psychological anger, we employed two subjective emotion indices: PANAS and STAXI. The Positive and Negative Affect Schedule (PANAS) [17] measures subjective emotions in two independent dimensions (positive and negative emotion terms). The PANAS has been widely used as a measure of the subjective experience of anger and cortical asymmetry activity in past studies [18], [19], [20]. We used the Japanese version of the PANAS [21], which is based on the 20-item English version containing positive and negative affect subscales. Due to the approach motivational component of anger, the PANAS can sometimes detect the positive affective component of anger [19], [22]. Several studies have created tailored questionnaires in order to assess the approach motivational component of anger [11], [18], [23], [24], [25], [26]. However, we utilized a standardized scale used both in western countries and in Japan to avoid translation problems when assessing the motivational component of anger. We employed the State-Anger scale in the State-Trait Anger Expression Inventory (STAXI) [27], [28] to measure the approach motivational component of anger (the intensity of anger as an emotional state at a particular time; e.g., I feel like hitting someone), and it has previously been used to assess the approach motivational component of anger [29].

We predicted two possible outcomes. One possibility was that an apology would eliminate the approach motivational component of anger thus eliminating asymmetric frontal brain activity; however, the apology would not extinguish the subjective experience of anger [12], [16]. The other possibility was that the apology would eliminate not only the approach motivational component of anger, but also the subjective experience of anger. In either case, we predicted that the asymmetry of frontal brain activity would be altered so long as the apology was effective. We were particularly interested in determining whether ANS activity would be affected by receiving an apology following an insult.

## Results

We conducted a four-way analysis of variance (ANOVA): 2 (Group: no-apology vs. apology)×2 (Gender: male vs. female)×2 (Period: baseline vs. insult)×2 (Asymmetry: left vs. right) for the EEG data. We also conducted a three-way ANOVA without asymmetry for the HR and SCL measures. However, neither the main effect of gender (*Fs*<1.69, *ps*>.18, *η^2^s*<.04) nor any of the interactions with gender (*Fs*<.14, *ps*>.71, *η^2^s*<.03) were significant among the three measures. Thus, the subsequent analyses excluded gender as a factor.

### EEG Results

**Figure 1:**
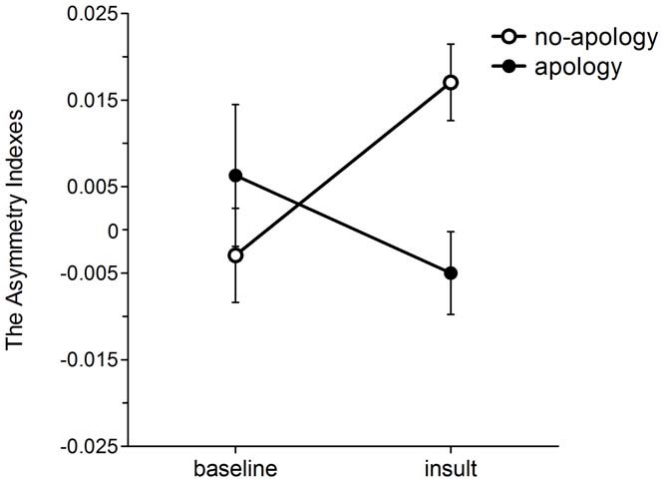
The asymmetry indexes for each group were displayed in the baseline and the insult periods. The open circles illustrate the no-apology group (*N* = 24). The closed circles illustrate the apology group (*N* = 24). Each vertical line illustrates the standard error for each condition.

As in previous studies [12], [16], [18], the alpha power values at each brain site (F7 and F8) were submitted to a natural log transformation to normalize the distributions. Next, asymmetry indices were calculated by subtracting from log (F8) to log (F7). The asymmetry scores are displayed in Figure 1.

Greater asymmetry in frontal brain activity was observed when participants (no-apology group) read the insulting sentences without the apology comment (Figure 1) but not when the participants (apology group) read the same sentences with the simple apology. A two-way ANOVA (Group×Period) revealed a significant interaction between Group and Period, *F*(1, 46) = 6.32, *p* = .015, *η^2^* = .12; however, the main effects of Group, *F*(1, 46) = 1.32, *p* = .256, *η^2^* = .028, and Period, *F*(1, 46) = .49, *p* = .488, *η^2^* = .011, were not significant. To deconstruct the significant interaction, post-hoc tests (*t*-test) were conducted in each group. The asymmetry index significantly increased in the no-apology group following the insulting comments, *t*(23) = 2.84, *p* = .009, *r* = .51, 95% confidential interval (CI) = −.03, −.007. However, there was no significant difference between the two periods in the apology group, *t*(23) = 1.10, *p* = .282, *r* = .22, 95% CI = −.009, .03. These results suggest that the simple apology reduced the approach motivation of anger.

It is possible that the apology “prevented” the increase in approach motivation or participants may have just displayed a quick recovery after approach motivation increased [2], [3], [5]. Detailed analyses suggest the former. The alpha power showed significant asymmetry for the no-apology group, *t*(23) = 5.03, *p* = .0001, 95% CI = −.012, .05, but not in the apology group during the earliest 30 seconds of the recording period, *t*(23) = 0.19, *p* = 2.62, 95% CI = −.18, .061. The HR response of the no-apology group also increased significantly in the earliest 30 seconds, *t*(23) = 3.97, *p* = .008, 95% CI = −8.0, −.15, whereas HR response of the apology group did not increase during the same period, *t*(23) = .17, *p* = .008, 95% CI = −.10, .15.

### ANS Results

**Figure 2:**
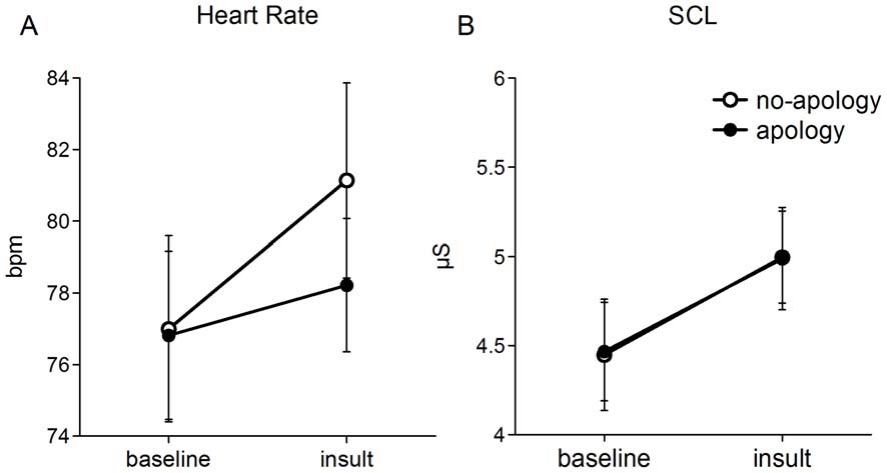
The results of HR (A) and SCL (B) for each group were displayed in the baseline and the insult periods. Each vertical line illustrates the standard error for each condition.

Figure 2 shows the results of the ANS measures in the two groups. The HR results showed a similar pattern to that of the EEGs (Figure 2A). When participants read the insulting comments, the HR response of the apology group increased sharply, whereas it increased mildly in the apology group. A 2 (Group: apology vs. no-apology)×2 (Period: baseline vs. insult) ANOVA yielded a main effect of Period, *F*(1, 46) = 7.73, *p* = .008, *η^2^* = .144; however, the main effect of Group, *F*(1, 46) = .28, *p* = .636, *η^2^* = .005, and the interaction, *F*(1, 46) = 1.89, *p* = .176, *η^2^* = .039, were not significant. Given the significant interaction in the EEG measure and our special interest in the ANS measures following the insult, we performed planned comparisons in each group. The HR response in the no-apology group increased significantly following the insult, *t*(23) = 3.28, *p* = .003, *r* = 0.56, 95% CI = −6.75, −1.53; however, the HR response of the apology group did not increase, *t*(23) = .91, *p* = .373, *r* = 0.19, 95% CI = −4.59, −1.79, suggesting a differential effect of a simple apology on HR reactivity.

Results of the SCL were different from the EEG and HR results (Figure 2B). Both groups showed increased SCL response following the insult. A 2 (Group: apology vs. no-apology)×2 (Period: baseline vs. insult) ANOVA revealed a main effect of Period, *F*(1, 46) = 17.91, *p* = .0001, *η^2^* = .280; however, the main effect of Group, *F*(1, 46) = .0002, *p* = .989, *η^2^* = .000004, and the interaction were not significant, *F*(1, 46) = .012, *p* = .913, *η^2^* = .0003. A planned comparison in each group showed a significant increase in the SCL in both groups (no-apology: *t*(23) = 3.01, *p* = .006, *r* = 0.53, 95% CI = −.89, −.16; apology: *t*(23) = 2.97, *p* = .007, *r* = 0.53, 95% CI = −.85, −.15), suggesting that the SCL measure was sensitive to the insult but was insensitive to the simple apology.

### Results of psychological anger

#### The results of psychological anger are summarized in Table 1

**Table 1:** Mean rating scores and standard error of subjective scales (PANAS and STAXI) for the no-apology group and the apology group.

	no-apology		apology	
	baseline	insult	baseline	insult
PANAS				
Positive	2.8 (0.2)	2.5 (0.2)	2.7 (0.2)	2.5 (0.3)
Negative	1.6 (0.1)	2.9 (0.2)*	1.8 (0.1)	3.1 (0.2)*
STAXI	1.1 (0.04)	1.6 (0.1)*	1.1 (0.03)	1.1 (0.1)

* :baseline<insult (*ps*<.05).

The no-apology group showed higher STAXI scores than the apology group, while both groups reported increased STAXI scores in the insult period. A 2 (Group: apology vs. no-apology)×2 (Period: baseline vs. insult) ANOVA confirmed these observations. The main effects of Group, *F*(1, 46) = 6.40, *p* = .015, *η^2^* = .122, and Period, *F*(1, 46) = 23.61, *p* = .00001, *η^2^* = .339, were significant. Importantly, the interaction was also significant, *F*(1,46) = 15.39, *p* = .00001, *η^2^* = .251. Post-hoc analyses revealed an increase in anger in the no-apology group, *t*(23) = 4.59, *p* = .0001, *r* = 0.69, 95% CI = −.73, −.28, but no increase in anger in the apology group, *t*(23) = 1.62, *p* = .120, *r* = 0.32, 95% CI = −.12, .02. These results suggest that the apology suppressed a subjective anger state as measured by the STAXI.

Results from the negative affect subscale of the PANAS were not different between the two groups. A two-way ANOVA revealed a main effect of Period, *F*(1, 46) = 39.24, *p*<.000001, *η^2^* = .631. However, the main effect of Group, *F*(1, 46) = .48, *p* = .490, *η^2^* = .010, and the interaction, *F*(1, 46) = .003, *p* = .957, *η^2^* = .00006, were not significant. A two-way ANOVA was also performed on the positive affect subscale of the PANAS. The main effect of Period was significant, *F*(1, 46) = 4.60, *p* = .037, *η^2^* = .091; but, again, the main effect of Group, *F*(1, 46) = .009, *p* = .924, *η^2^* = .0002, and the interaction, *F*(1, 46) = .323, *p* = . 572, *η^2^* = .007, were not significant.

The STAXI results seem to correspond with the EEG and HR results. Conversely the results of the negative affect subscale of the PANAS seemed to correspond with those of the SCL. Therefore, we examined correlations between the negative affect subscale scores from the PANAS and the physiological measures in the two groups. There was no significant correlation between the negative affect subscale and the SCL measure (no-apology: *r* = .004, *N* = 24, *p* = .986; apology: *r* = −.003, *N* = 24, *p* = .989). There was also no significant correlation between the STAXI and the HR measure (no-apology: *r* = −.182, *N* = 24, *p* = .394; apology: *r* = −.233, *N* = 24, *p* = .273).

## Discussion

In the present study, an apology eliminated the asymmetry in frontal brain activity and but influenced an increase in HR reactivity; however, the apology did not affect changes in SCL reactivity in response to an anger provocation. Previous studies have shown that asymmetrical frontal brain activity reflects the approach motivational component of anger [8], [9], [11], [12], [13], [14]. The simple apology used in this study also successfully suppressed the approach motivational component of anger. However, the restricted efficacy of the apology was evident in the psychological scales. Studies have shown that the prevention of the approach motivation reduces the asymmetry of frontal brain activity but it does not necessarily reduce the subjective experience of anger [12], [16]. Consistent with these findings, an anger state relevant to the approach motivation (via the STAXI) was reduced by the apology, whereas subjective scores of negative emotion (via the PANAS) were not altered either with or without the apology. These results correspond with the participants' introspective reports: no one was soothed by the apology in this study. Therefore, our results suggest that, the simple apology had little effect on calming down the experience of anger.

The most interesting finding was that the HR and the SCL reactivity showed different susceptibility to the apology. Several studies examining anger have demonstrated that both HR and the SCL reactivity increase when people get angry [4], [30]. This was the case in the present study for the no apology group. However, the HR and the SCL responses are assumed to reflect different components of anger. The HR reactivity as well as the asymmetrical frontal brain activity is assumed to reflect the approach motivational component, whereas SCL responses reflect the negative emotional component of anger (see Figure 2 and Table 1). This view is consistent with the distinction between anger and fear. An increase in SCL response is observed when people experience both anger and fear [4], [30]. Conversely, HR reactivity typically does not increase when experiencing fear [4], [30]. Both fear and anger produce the negative emotional component, which corresponds to an increase in SCL reactivity. The major distinction between anger and fear is the approach motivation component. In the case of anger, HR reactivity increases, while this is not the case for fear. The present study suggests that anger is not a unitary process as a basic emotion but has multiple components that can be measured through different physiological activities. Further examination will be needed to clarify how these ANS measures change in response to an insult and the following apology. This is important given that HR activity has a complex relationship with emotion, motivation, and attention. Thus, increased HR reactivity might not necessarily reflect approach motivation, alone.

Why are apologies ubiquitous all over the world despite having such a limited effect on anger? When aggression [11], [13], [31] or approach motivation [12], [16], [32] is suppressed, frontal brain asymmetry has been eliminated in response to anger provocation. An apology may be efficacious in suppressing the asymmetry of brain activity (e.g., the approach motivation of the angry person), which may help people avoid being the victim of anger. The apology may allow the person giving the apology to avoid a violent outburst from the angry person; however, this may not eliminate the experience of negative emotion for the angered person.

In summary, the present study clearly showed that anger is not a unitary process but has multiple components that appear as different physiological reactions to an apology. An apology may eliminate the approach motivational component of anger without affecting the subjective experience of anger as measured by SCL.

## Materials and Methods

### Participants

Forty-eight students (female = 24, mean age = 20.5) from a local university participated in the experiment. All participants were right-handed, as assessed by the Edinburgh Handedness Inventory [33], and had normal or corrected-to-normal vision according to a self-report. All participants were naive to the purposes of the experiment and gave their written informed consent. The Ethics Committee of the Japan Science and Technology Agency approved the experimental protocol.

### Procedure

The participants came to the laboratory under the assumption that there was another participant in another experimental room. The experimenter was careful to drop subtle hints during the course of the experiment to make this cover story believable. Participants were told this experiment would record EEG and ANS when they are discussing social problems (e.g., a tuition hike, smoking in public) by exchanging their brief, hand-written opinions.

After obtaining consent, EEG and ANS sensors were attached to the participants. Then, during a 2 min rest period, baseline EEG and ANS data were recorded. After the baseline recording, participants completed the PANAS and the STAXI questionnaires. Next, participants were told they had been randomly assigned to write an essay and that the other participant would evaluate it. The participants were given 10 min to write the essay. The essay was then brought to the other fictitious participant for evaluation, while the participants received a handwritten essay by the fictitious counterpart and asked to evaluate it. The evaluation included ratings of the essay on six characteristics using a 9-point scale (e.g., for intelligence, 1 = unintelligent, 9 = intelligent). In addition, there was a comment column on the evaluation sheet where the participants were required to provide a comment about the counterpart's essay. The evaluation by the fictitious counterpart was then returned to the participants. All participants were given the following ratings: intelligence = 3, interest = 3, friendliness = 2, logic = 3, respectability = 4, and rationality = 3. Each essay was also provided with this comment: “I can't believe an educated person would think like this. I hope this person learns something while at university.” [11]. A female handwrote all of the feedback. This insult manipulation has been successfully used in prior studies [11], [16]. However, there were extra comments provided in the present experiment. For the no apology group, the second comment said, “That is all of my comments.” For the apology group, the second comment said, “I'm sorry for making such a critical comment on your essay.” were added to the end of the above insulting sentence as the experimental manipulation. The participants were required to read the feedback ratings and comments silently for 2 min while EEG and ANS data were recorded. They next filled out the subjective emotional questionnaires (PANAS and STAXI) for a second time. Participants were debriefed at the end of the experiment.

### Recordings

EEG signals were recorded from lateral frontal sites (F7, F8 according to the 10–20 system) using Ag/AgCl electrodes. The ground electrode was mounted at the midline between the frontal pole and the frontal site. The reference electrode was placed at the tip of the nose. Vertical eye electrooculograms (EOGs) were also recorded to facilitate artifact correction of the EEG. All electrode impedances were under 5 kΩ. The sampling rate of the EEG was 500 Hz. EEGs and EOGs were amplified with an MP150 data acquisition system (BIOPAC Systems, Inc., Goleta, CA), a digital bandpass filter of (0.5–30 Hz) was applied, and ocular artifacts were corrected using the method described in a previous study [34]. Alpha power was calculated by fast Fourier transform using a Hamming window within the alpha band (8–13 Hz). Because alpha power is inversely related to cortical activity, higher alpha power on the right side than the left side indicates greater activity in the left than the right [15], [35].

HR and SCL were recorded by an MP150 system (BIOPAC Systems, Inc., Goleta, CA). HR was recorded from the left and right wrists by disposable electrodes. From the 2-min baseline data and the silent reading of the insult sentence, the beats per minute (bpm) were extracted using Acknowledge software (BIOPAC Systems, Inc., Goleta, CA). For the SCL recordings, two sweat-isotonic electrodes were placed on the palmar sites of the middle phalanges of the second and third fingers of the left hand [36]. SCL was calculated by averaging the skin conductance response amplitude in the 2 min baseline period and during the silent reading of the insult sentence. Both ANS measures were sampled at 500 Hz.

Subjective affect scales were administered after recording psychophysiological data during the baseline and insult periods. We used two measures as a subjective scale to assess anger, the PANAS and STAXI. The PANAS is an inventory of a participant's mood on a 7-point scale (1 = very slightly, 7 = extremely) to positive/negative items [17]. The present experiment used the Japanese version of the PANAS [21], which was based on the original PANAS. The STAXI [27], [28] was also used as a subjective scale for anger in the present experiment. We used 10 items of state-anger in the Japanese STAXI to assess the state anger of the participants on a 4-point scale (1 = almost never, 4 = almost always). The participants also completed both scales after they read the comments provided by the insult manipulation.
